# Blo t 2: Group 2 allergen from the dust mite *Blomia tropicalis*

**DOI:** 10.1038/s41598-019-48688-y

**Published:** 2019-08-22

**Authors:** Kavita Reginald, Sze Lei Pang, Fook Tim Chew

**Affiliations:** 1 Department of Biological Sciences, Sunway University, Bandar Sunway 47500, Selangor, Malaysia; 2 Allergy and Molecular Immunology Laboratory, Department of Biological Science, National University of Singapore, Singapore, 117543 Singapore

**Keywords:** Proteins, Applied immunology

## Abstract

*Blomia tropicalis* has been recognized as a cause of allergic diseases in the tropical and subtropical regions. Here we report the immuno-characterization of its group 2 allergen, Blo t 2. Allergen Blo t 2 was amplified from the cDNA of *B. tropicalis* using degenerate primers, expressed in *Escherichia coli* as a recombinant protein and purified to homogeneity. The mature protein of Blo t 2 was 126 amino acids long with 52% sequence identity to Der p 2 and apparent molecular mass of 15 kDa. Circular dichroism spectroscopy showed that Blo t 2 is mainly a beta-sheeted protein. We confirmed the presence of three disulfide bonds in recombinant (r) Blo t 2 protein using electrospray mass spectrometry. Thirty-four percent of dust-mite allergic individuals from the Singapore showed specific IgE binding to rBlo t 2 as tested using immuno dot-blots. IgE-cross reactivity assays showed that Blo t 2 had between 20–50% of unique IgE-epitopes compared to Der p 2. IgE binding of native and recombinant forms of Blo t 2 were highly concordant (r^2^ = 0.77, p < 0.0001) to rBlo t 2. Dose-dependent *in vitro* histamine was observed when rBlo t 2 was incubated with whole blood of Blo t 2 sensitized individuals, demonstrating that it is a functional allergen. Nine naturally occurring isoforms of Blo t 2 were identified in this study, each having between 1–3 amino acid variations compared to the reference clone. Blo t 2 is a clinically relevant allergen of *B. tropicalis* as it has unique IgE epitopes compared to major group 2 allergens from *Dermatophagoides spp*.

## Introduction

Dust mites are an important source of indoor allergens worldwide, causing a range of allergic manifestations such as asthma, allergic rhinitis and atopic dermatitis^1^. Taxonomically, house dust mites belong to the phylum Arthropoda, subphylum Chelicerata, class Arachnida, order Acari, and suborder Astigmata^2^. From the 57 families in this suborder, the clinically important dust mites identified so far are restricted to only five families, namely Pyroglyphidae (genus *Dermatophagoides* and *Euroglyphus*), Echimyopodidae (genus *Blomia*), Acaridae (genus *Acarus*, *Tyrophagus* and *Aleuroglyphus*), Suidasiidae (genus *Suidaisia*) and Glycyphagidae (genus *Glycyphagus* and *Lepidoglyphus*). Of these, dust mites of the *Dermatophagoides* genus has been the most extensively characterized owing to its worldwide prevalence, and the potency of its allergens.

In contrast, the habitat of *Blomia tropicalis* is limited to the tropical and subtropical countries^3–10^. Dust-mite sensitized individuals living in these countries show varying degrees of skin prick reactivity to crude protein extracts of *B. tropicalis*, ranging from over 90% among individuals in Singapore, circa 70% among individuals in China and Taiwan to as low as 38% in Florida^9^. In terms of individual allergens, while group 1, 2 and 23 allergens are the major allergens of the *Dermatophagoides*-sensitized individuals^11,12^, IgE reactivity among *Blomia-*sensitized individuals are mainly directed against Blo t 5 and its paralogous counterpart Blo t 21^13–16^.

Group 2 dust mite allergens are some of the most widely studied and, to date, twelve different group 2 allergens have been identified^17–23^ of which Der p 2 and Der f 2 exhibit the highest degree of IgE binding. In this study, we report the identification and characterization of Blo t 2, in terms of its IgE-reactivity and cross-reactivity within the Singaporean population, histamine release capacity, presence in the indoor environment, and the frequency of naturally occurring isoforms of Blo t 2.

## Results

### Cloning and full-length sequencing of Blo t 2

**Figure 1:**
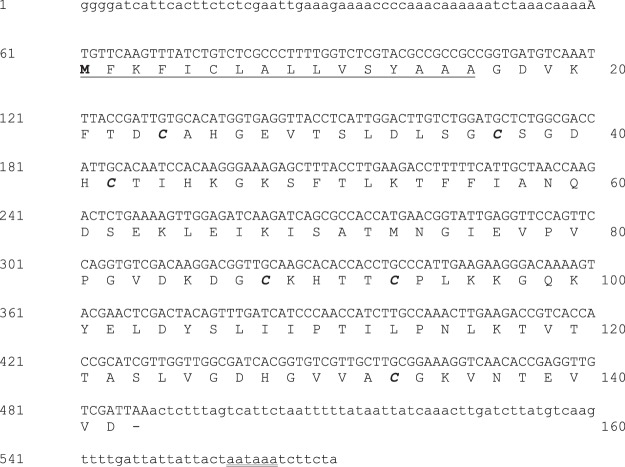
Nucleotide and translated amino acid sequence of Blo t 2. The DNA sequences in uppercase represent the open reading frame, while the sequences in lowercase are the untranslated sequences. The predicted initiation Met start codon is in bold face and underlined, and the polyadenylation signal is double underlined. The signal peptide of Blo t 2 is underlined. Cysteine residues along the predicted mature protein sequence are italicized and bold faced. The stop codon (TAA) is represented by a dash. The ML domain spans from V19 to E139 of the full-length sequence.

We amplified the group 2 allergen from *B. tropicalis* using a combination of two techniques, reverse transcription polymerase chain reaction (RT-PCR) and RACE (Random Amplification of cDNA Ends). First, RT-PCR amplification of total *B. tropicalis* RNA was performed using degenerate primers that were designed based on the conserved sequences of known group 2 allergens. A single amplicon band of 250 base pairs was observed on the agarose gel electrophoresis. Subsequently, specific primers were designed based on sequence of this short fragment to amplify the 5′ and 3′ ends of the full-length open reading frame by RACE. The full-length clone obtained was sequenced to confirm its identity. A homology search was performed using the sequence obtained against the non-redundant database in NCBI using the BLAST-X algorithm^24^, which showed a match to group 2 dust mite allergens, with the closest homology being to Ale o 2 (from *Aleuroglyphus ovatus*), with 60% amino acid sequence identity. This clone was named Blo t 2 (GenBank accession number DQ677253), in accordance with the nomenclature guidelines of the WHO/IUIS Allergens Nomenclature Subcommittee, Switzerland^25^. The complete clone of Blo t 2 had an open reading frame of 426 nucleotides, coding for 142 amino acid residues (Fig. 1).

Blo t 2 had a predicted signal peptide of 16 amino acids identified using the SignalP program^26^, and belonged to the MD-2-related lipid-recognition (ML) domain family^27^, similar to known group 2 allergens as identified by the Pfam domain search^28^. As with other published group 2 allergens, the mature sequence of Blo t 2 also has 6 cysteine residues (Fig. 1, Supplementary Figs 1 and 2). The presence of these cysteine residues in recombinant Blo t 2 (rBlo t 2) has been confirmed using a combination of tryptic digestion and mass spectrometry (Supplementary Fig. 3A,B). Previous reports have shown that the cysteine residues in group 2 allergens form disulfide bonds and are important for their structural integrity and allergenicity^29,30^.

### Expression, purification and biochemical characterization of rBlo t 2

**Figure 2:**
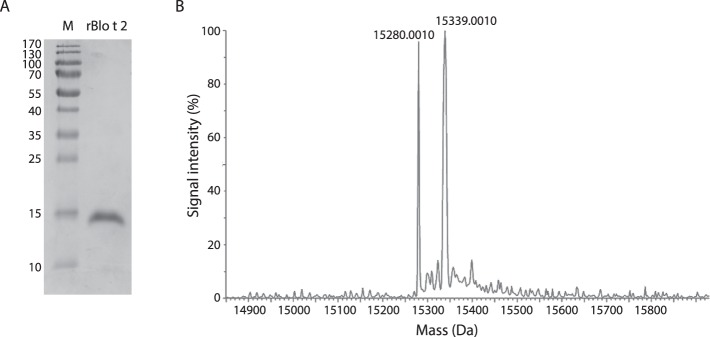
Analysis of purified recombinant Blo t 2 (rBlo t 2) (**A**) Purified rBlo t 2 and molecular mass marker (M) was separated by 15% SDS-PAGE and stained with Coomasie brilliant blue. (**B**) ESI-TOF mass spectra of rBlo t 2. Mass of rBlo t 2 protein, both with (15,280 Da) and without modification (15,339 Da) are indicated on top of each peak.

The Blo t 2 gene was cloned into a modified pET-32a(+) vector (pET-M) and overexpressed as a 6× His-tag fusion protein. The rBlo t 2 protein was purified to homogeneity using Ni-NTA affinity chromatography, followed by size-exclusion chromatography. The SDS-PAGE analysis showed the molecular weight of purified rBlo t 2 to be approximately 15 kDa (Fig. 2A, Supplementary Fig. 7). The identity of rBlo t 2 by mass spectrometry was confirmed using the TripleTOF 5600 system (SCIEX), with 95.8% of the protein sequences matching that of rBlo t 2 (Supplementary Fig. 3; Supplementary Table 1). More than 84 percent of the protein sequence also matched sequences of the Blo t 2 isoforms (UniProt ID: ABG76188.1, ABG76190.1, ABG76193.1, ABG76191.1, and AAQ73482.1).

When rBlo t 2 was subjected to electrospray mass spectrometry, it revealed a molecular mass of 15,280Da (Fig. 2B). However, the calculated (theoretical) molecular weight of rBlo t 2 using the ProtParam tool^31^ was 15,286Da. The difference of 6Da between the calculated and experimental molecular mass of rBlo t 2 corresponds to the loss of 6 hydrogen atoms during the formation of three disulfide bonds.

The electrospray mass spectrum also shows an additional peak at 15,339Da which corresponds to the carbamylation and oxidation post translational modifications of rBlo t 2 that most likely occurred during sample preparation. Carbamylation and oxidation caused the addition of 43 and 16Da, respectively (Fig. 2B).

Recombinant Blo t 2 displayed a typical β-sheeted structure, as determined by circular dichoism analysis (Supplementary Fig. 4A). In addition, the predicted structure of Blo t 2 based on the solved structure of Der f 2 (PDB ID: 2F08) superimposed with the crystal structure of Der p 2 (PDB ID: 1KTJ) with a low root mean square deviation (r.m.s.d) of 1.45 Å, signifying that Blo t 2 likely shares the same three-dimensional structure as Der p 2^30^ and Der f 2^32–34^ (Supplementary Fig. 4B).

### Allergenicity of Blo t 2

**Figure 3:**
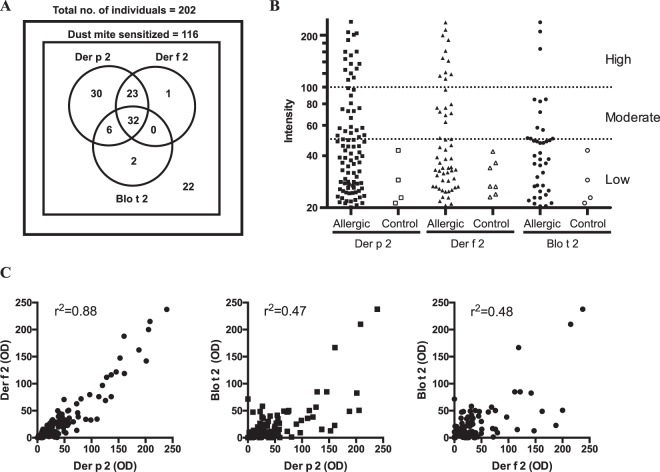
(**A**) Venn diagram depicting the number of individuals with positive IgE-reactions to recombinant Der p 2, Der f 2 and Blo t 2 as measured using immuno-dot blots. (**B**) Scatter plot of specific IgE-binding of dust-mite allergic individuals (n = 116) and control (n = 86) (atopic, but not allergic to dust mite extracts) individuals to recombinant Der p 2, Der f 2 and Blo t 2. The amount of IgE-binding was measured as optical density. (**C**) Dot-plot of IgE-binding of atopic patients’ sera to Der p 2, Der f 2 and Blo t 2, showing the IgE-binding intensities and correlation between allergens. R square values of linear regression was indicated (p < 0.0001).

Specific IgE reactivity to Blo t 2 in comparison to those of Der p 2 and Der f 2 were assayed by immuno dot-blot assays using sera from a total of 202 allergic sera from Singapore. Of these, 116 sera were dust-mite sensitized individuals with IgE binding to crude dust mite extracts of *Dermatophagoides pteronyssinus* and *Blomia tropicalis* species which are predominant in the indoor environment of Singapore^3^. Thirty-four percent of dust-mite positive individuals showed IgE reactions to Blo t 2, while reactions to Der p 2 and Der f 2 were 78% and 48% respectively (Fig. 3A,B). The amount of IgE binding to each allergen was quantified based on the intensity of the IgE reaction. Among the Singaporean individuals with IgE reactivity to Der p 2, 16 showed high reaction (intensity > 100, equivalent to Class 3 specific IgE levels), 18 showed moderate reactivity (50 < intensity < 100, equivalent to Class 2 specific IgE levels) while the remaining had low specific IgE reaction (20 < intensity < 50, equivalent to Class 1 specific IgE levels) (Fig. 3B). For Der f 2, 11 of the 56 Der f 2 positive patients showed high reaction, while 9 showed moderate reaction. In contrast, only three dust-mite sensitized individuals showed high reactivity to Blo t 2, and nine with moderate IgE-reactions (Fig. 3B). Control individuals mostly did not show specific IgE reactivity to the three recombinant allergens tested, although a few (n ≤ 7) had low specific IgE reaction (equivalent to Class 1 specific IgE levels) which could be due to cross-reactivity to other allergens (e.g. group 2 allergen homologues from storage mites^22^).

The concordance of IgE reaction between the group 2 allergens were assessed based on the Spearman’s rank correlation values. IgE reactivity between Der p 2 and Der f 2 (both belonging to the pyroglyphid mite family) were highly concordant (r^2^ = 0.88, p < 0.0001) (Fig. 3C). However, only moderate concordance was observed for IgE reactions between Der p 2 and Blo t 2 (r^2^ = 0.47, p < 0.0001), or Der f 2 and Blo t 2 (r^2^ = 0.48, p < 0.0001).

**Figure 4:**
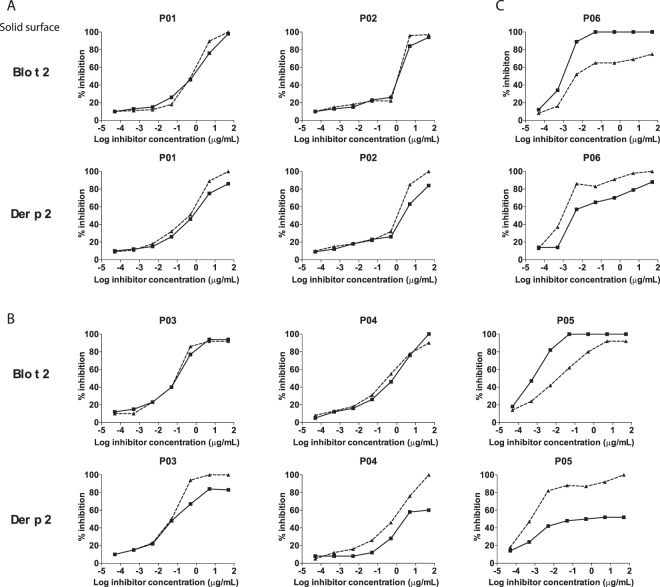
Competitive IgE ELISA assay. Six individual sera with different reaction profiles to Der p 2 and Blo t 2 (**A**) high IgE-reaction to both allergens; (**B**) high IgE reaction to Der p 2, moderate IgE reaction to Blo t 2; and (**C**) moderate IgE reaction to Blo t 2, low IgE reaction to Der p 2 were tested. Sera were pre-absorbed with various concentrations of inhibitors before incubation with Der p 2 or Blo t 2 in coated plates (solid surface). Solid lines indicate sera has been incubated with Blo t 2, whereas dotted line indicates sera has been incubated with Der p 2. The amount of inhibition was calculated in relation to the uninhibited control.

As concordance of IgE reactions between two allergens could arise from either cross-reactivity or co-sensitization (dual sensitization), an inhibition ELISA assay was used to evaluate the presence of cross-reactivity between the allergens tested using sera from selected individuals with different IgE reaction profiles to Der p 2 and Blo t 2 (Fig. 4, Supplementary Fig. 5).

Two individuals (P01 and P02) with high IgE reactivity to both Der p 2 and Blo t 2 showed complete self-inhibition to Blo t 2, and cross-inhibition to Der p 2 when Blo t 2 was used in the solid phase (Fig. 4A, upper panel). In these same patients, a maximum of about 80% cross-inhibition was observed for Blo t 2, when Der p 2 was used in the solid phase (Fig. 4A, lower panel). In contrast, three patients (P03, P04 and P05), having high IgE reactivity to Der p 2 but moderate reactivity to Blo t 2 showed a lower amount of cross-inhibition (between 50–80%) between both allergens, signifying that 20–50% of the epitopes were unique between both allergens (Fig. 4B). One patient (P06) who showed moderate IgE-reactivity to Blo t 2 but low IgE-reactivity to Der p 2, the inhibition profile showed that both allergens were not able to completely inhibit the other (Fig. 4C). For P06, unique and cross-reactive epitopes of both Der p 2 and Blo t 2 seemed to influence this patient’s IgE reactivity.

### Isolation of native Blo t 2

**Figure 5:**
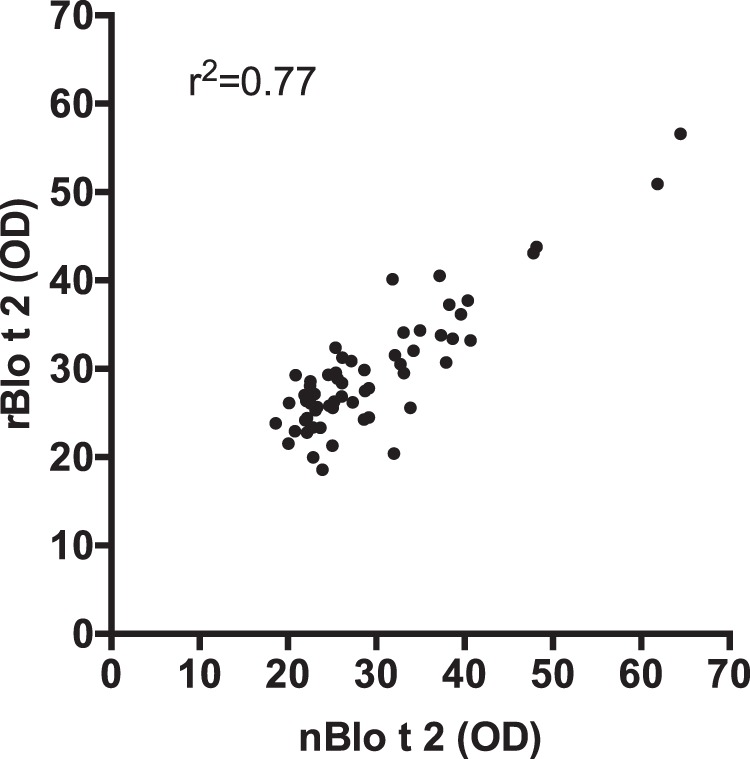
Correlation between the amount of IgE binding of 59 dust-mite allergic individuals’ sera to native Blo t 2 (nBlo t 2) and recombinant Blo t 2 (rBlo t 2). The amount of IgE binding is represented by optical density (OD) of the immuno-dot blot colorimetric reaction. Linear regression analysis shows that the IgE binding to the native and recombinant Blo t 2 were correlated (r^2^ = 0.77, p < 0.0001).

The native protein of Blo t 2 (nBlo t 2) was isolated from the total protein extract of *B. tropicalis* using an antibody pull-down method. Both nBlo t 2 and rBlo t 2 were then tested for IgE binding in 59 dust-mite allergic individuals using IgE dot blot assay. The IgE binding to rBlo t 2 was highly concordant to its native Blo t 2 (nBlo t 2) (r^2^ = 0.77, p < 0.0001) (Fig. 5), suggesting that both proteins had comparable allergenicity.

### Histamine release of Blo t 2

**Figure 6:**
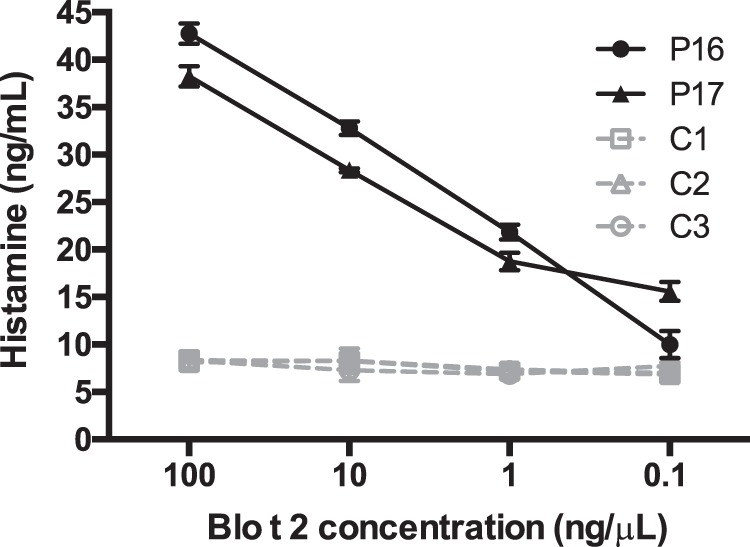
*In vitro* histamine release from Blo t 2. Two hundred microliters of whole blood from two Blo t 2 allergic individuals and three non-allergic controls were challenged with serially diluted Blo t 2 for one-hour to stimulate histamine release. The amount of histamine released was measured by an ELISA assay. Mean and standard errors of duplicate experiments are shown.

Whole blood of two dust-mite allergic individuals, P16 and P17 were collected, and incubated with serial dilutions of recombinant Blo t 2 to stimulate *in vitro* histamine release, as measured by ELISA. Both individuals tested displayed dose-dependent histamine release upon stimulation with rBlo t 2 (Fig. 6). When control individuals (n = 3) were tested for histamine release with serially diluted Blo t 2, the amount of histamine measured were within the reference range for whole blood^35^, averaging at 8.25 ng/mL, indicating the absence of non-specific histamine release at any Blo t 2 concentrations tested (Fig. 6).

### Blo t 2 is present in the environmental dust samples

**Figure 7:**
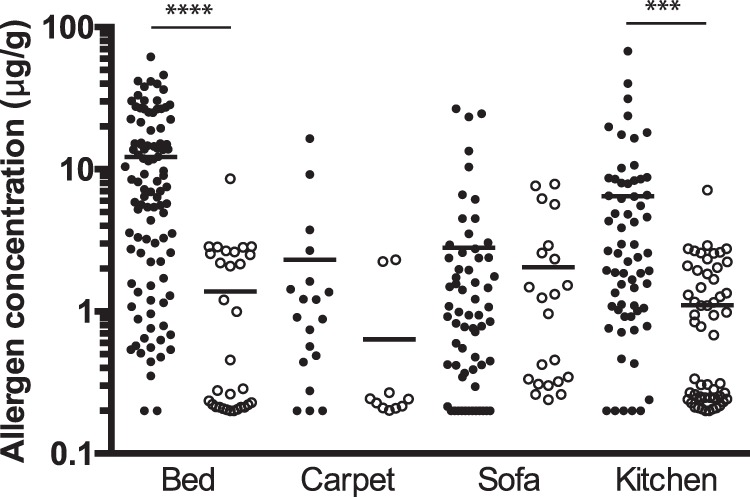
Concentration of Blo t 2 and Der f 2 in dust samples from Singaporean homes. Dust samples were collected by vacuuming four areas within a home and assayed for the amounts of Blo t 2 (●) and Der f 2 (○) in beds (n = 101; 31), carpets (n = 19; 10), kitchens (n = 63; 22) and sofas (n = 65; 56). Horizontal bars on the scatter plot show the geometric mean concentration for each allergen. Asterisks denotes p-value of statistically significant differences; (****)p < 0.0001, (***)p < 0.001.

Next, the concentration of Blo t 2 in the dust samples from Singaporean homes were measured (Fig. 7). Dust samples were collected by vacuuming 4 areas within a home; bed, carpet, kitchen and sofa, and the amounts of allergen present were assayed by ELISA. The concentrations of Blo t 2 were compared with that of Der f 2, to investigate the presence of a link between the levels of allergen in the environment. The average concentration of Blo t 2 measured was the highest in beds (12.2 μg/g dust) and lowest in carpets (2.3 μg/g dust), whereas for Der f 2, the highest concentration as detected in the kitchens (2.0 μg/g dust) and the lowest in carpets (0.64 μg/g dust) (Fig. 7). Of the four niches studied, the levels of Blo t 2 was only significantly higher than Der f 2 in beds (p < 0.0001) and sofa (p = 0.0002). On average, the concentration of Blo t 2 was approximately 4-fold higher than that of Der f 2 in all niches (Fig. 7).

### Isoforms of Blo t 2

**Figure 8:**
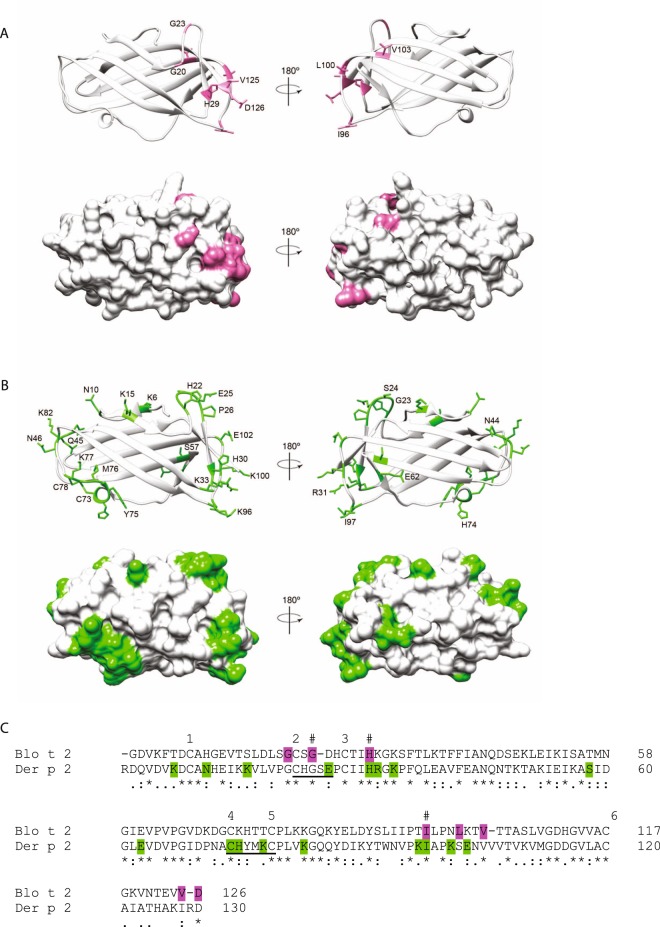
(**A**) Polymorphic amino acid residues (colored pink) of the different Blo t 2 isoforms were mapped and shown as both ribbon and space filled models in two different orientations. The homology model of Blo t 2 was deduced using the SWISS-MODEL server based on the crystal structure of Der f 2 (Protein data bank ID: 2F08) as a template^69^. **(B**) The IgE epitope residues of Der p 2 (colored green) (listed in Supplementary Table 2) were mapped on the solved structure of Der p 2 (PDB ID: 1KTJ) and shown as both ribbon and space filled models in two different orientations. (**A**,**B**) were generated using Chimera^70^. (**C**) Comparison of IgE epitope residues of Der p 2, and polymorphic residues of Blo t 2. Multiple alignment of mature proteins of Blo t 2 (GenBank ID ABG76185) and Der p 2 (AAF86462) was performed using Clustal O (v1.2.4). IgE epitopes of Der p 2 were highlighted green while sequences which were deleted or mutated in tandem are underlined and correspond to the residues listed in Supplementary Table 1. Polymorphic residues of Blo t 2 were highlighted pink and correspond to the residues listed in Tables 1 and 2. The pound sign (#) indicates amino acid residues in Blo t 2 that is likely to be an IgE epitope due to similarity with experimentally confirmed IgE epitopes on Der p 2 (Supplementary Table 1). An asterisk (*) indicated the positions with fully conserved residues, a colon (:) indicated conservation between groups of strongly similar properties, and a period (.) indicated conservation between groups of weakly similar properties. Numbers above the sequences indicate the presence of a cysteine residue in any of the proteins.

Isoforms are a common feature of group 2 allergens^36–38^. In order to identify naturally occurring polymorphic forms of Blo t 2, seventy-four *E. coli* colonies containing the Blo t 2 insert were randomly selected and sequenced. Nine isoforms of Blo t 2 were identified based on the translated mature protein sequence of Blo t 2 and were named according to the allergen nomenclature guidelines (WHO/IUS Allergen Nomenclature Subcommittee World Health Organization, 1994) (Tables 1 and 2). Two nucleotide changes were observed in the signal peptide region of Blo t 2, at 2 and 12 amino acids upstream of the signal peptide cleavage site (Table 1, Fig. 1). Polymorphisms within the mature protein of Blo t 2 resulted in eight amino acid residue changes, with a slight heterogeneity in terms of the isoelectric point (pI) between Blo t 2 isoforms (Table 2). For one residue, V125 two outcomes of polymorphisms (V125F or V125I) was observed in Blo t 2.0105 and Blo t 2.0109 respectively (Table 2). All isoforms had at least one amino acid change, with a maximum of 3 amino acid substitutions for Blo t 2.0108 (Table 2). Blo t 2.0101 was the isoform with the highest frequency of 82.4%, followed by Blo t 2.0102 with 8.1% frequency. In three of the 9 isoforms identified, changes in the property of the amino acid residues were observed. In Blo t 2.0104, D126A caused a switch from charged polar to a non- polar residue, whereas for Blo t 2.0107 and Blo t 2.0108, polymorphisms in G23D and G20R respectively caused a change from non-polar to charged polar residues. All polymorphic residues of Blo t 2 were solvent exposed and clustered in the same region of which 7 of the 8 residues were situated at the loop region, and one residue was situated on the β-strand (Fig. 8A).

**Table 1:** Nucleotide and amino acid changes in isoforms of Blo t 2.

Amino acid residue no. (mature protein)	Blo t 2.0101	Polymorphic form
−12	ATC (I)	ATT (I)
−2	GCC (A)	ACC (T)
20	GGA (G)	AGA (R)
23	GGC (G)	GAC (D)
29	CAC (H)	CGC (R)
32	AAG (K)	AAA (K)
96	ATC (I)	GTC (V)
100	TTG (L)	GTG (V)
103	GTC (V)	ATC (I)
125	GTC (V)	ATC (I); TTC (F)
126	GAT (D)	GCT (A)

Codon and amino acid replacements (in parentheses) for each polymorphic form are shown. The nucleotide changes are underlined.

**Table 2:** Amino acid polymorphism in the isoforms of Blo t 2 is shown using Blo t 2.0101 as the reference clone.

Isoforms	Reference clone	Polymorphism	Frequency (of 74)	Percent frequency (%)	pI
Blo t 2.0101	Blo t 2	—	61	82.43	5.91
Blo t 2.0102	Blotn11	I96V, L100V	6	8.11	5.91
Blo t 2.0103	Blotn38	H29R	1	1.35	6.07
Blo t 2.0104	Blotn52	D126A	1	1.35	6.20
Blo t 2.0105	Blotn56	V125F	1	1.35	5.91
Blo t 2.0106	Blotn71	V103I	1	1.35	5.91
Blo t 2.0107	Blotn75	G23D	1	1.35	5.66
Blo t 2.0108	Blotn88	G20R, I96V, L100V	1	1.35	6.20
Blo t 2.0109	Blotn128	V125I	1	1.35	5.91

The frequency of polymorphic clones corresponding to each isoform is reported (n = 74). The theoretical isoelectric point (pI) of each isoform is listed as calculated by the ProtParam tool (ExPASy)^31^.

In order to evaluate if the polymorphisms observed for Blo t 2 could influence IgE binding, we compared the positions of the polymorphic residues of Blo t 2 to the published epitopes of Der p 2 (Fig. 8B,C, Supplementary Table 2). Blo t 2 polymorphisms involving residues G23 (Blo t 2.0107), H29 (Blo t 2.0103) and I96 (Blo t 2.0102 and Blo t 2.0108) aligned perfectly with residues reported to be important IgE epitopes in Der p 2^39–43^ (Fig. 8C, Supplementary Table 2), hence it is possible that polymorphisms in these Blo t 2 isoforms could have some impact on its IgE binding. However, further validation would be required to obtain a better understanding the implication of these polymorphisms and differences of pI on the IgE binding capacity of Blo t 2.

## Discussion

Blo t 2 demonstrates the typical characteristics of group 2 allergens of six cysteine residues, a signal peptide, and its recombinant protein (rBlo t 2) refolded to form beta-sheeted structures, similar to solved structures of Der p 2 and Der f 2^30,32–34^. The identity of and formation of three disulfide bonds in rBlo t 2 was confirmed using mass spectrometry analyses. The protein sequence of Blo t 2 also contained the ML domain, which is implicated in lipid recognition^27^. Recently, we have demonstrated that cholesterol is the preferred lipid ligand of Der p 2^44^, but further studies are necessary to identify the lipid ligand and its functional role in Blo t 2.

*Blomia tropicalis* is an important source of indoor allergens the tropical and subtropical countries. In our study, about 80% of the adult dust-mite sensitized individuals tested from Singapore had specific IgE-reactivity to Der p 2, with 34% reacted specifically to Blo t 2. In an earlier report by Kidon *et. al*., the prevalence of Blo t 2 was reported to be approximately 18%^45^, and this difference could be attributed to the fact that the subjects tested were much younger (mean age 7.3 years), whereas in the present study the subjects are adults (mean age 24 years). Similarly, a study by Posa *et. al*. reported that the IgE repertoire to twelve *D. pteronyssinus* allergens increased as the allergic patients aged^46^. The increase in IgE repertoire could be attributed to the epitope spreading phenomenon, where an antibody response to one epitope leads to production of new antibodies to other epitopes in the same or unrelated allergen^47^.

The majority of dust-mite sensitized individuals in our cohort showed high IgE binding to Der p 2 but moderate to low IgE reactivity to Blo t 2. Data from our IgE-cross reactivity studies showed that Blo t 2 had between 20-50% of unique IgE epitopes when compared to Der p 2. For other *B. tropicalis* allergens that have been characterized such as Blo t 1^48^, Blo t 5^49^, Blo t 7^50^, and Blo t 21^51^, low to moderate cross-reactivity between IgE epitopes of the *Blomia* allergens when compared to their *Dermatophagoides* homologues. These observations are expected in view of the large evolutionary distance between both mite species (Supplementary Fig. 6).

IgE reactivity to native Blo t 2 isolated from the crude protein extract of *B. tropicalis* was highly concordant to recombinant Blo t 2, suggesting that the recombinant allergen was immunologically similar to its native counterpart. For an allergen to cause an allergic reaction, it must be able to bind to IgE and cross-link IgE antibodies bound to mast or basophil cells, causing the cells to degranulate and release inflammatory mediators. Recombinant Blo t 2 was able to elicit dose dependent histamine from whole blood of two dust-mite allergic individuals, suggesting that it is capable of IgE cross-linking.

Exposure to greater than 2μg of Der p 1 and/or Der f 1 per gram of dust has been shown to be a risk factor for sensitization to mites and bronchial hyper-reactivity^52,53^. In the indoor environment, the average concentration of Blo t 2 was found to be about 4 fold higher that of Der f 2 in Singaporean homes and was well over the minimum threshold value of 2 μg/g dust to cause sensitization. While there was much lesser Der f 2 detected in the indoor environment, there were more patients that showed specific IgE reaction to Der f 2, and at higher intensity of reaction compared to Blo t 2. Hence, the relationship between the level of allergen present in the environment and its allergenic potency is not clear at present.

There have been several reports on the identification of group 2 allergen isoforms. In Der p 2, thirteen isoforms have been identified with frequent polymorphisms at positions 40, 47, 111 and 117^54^. The isoforms of Der p 2 did not vary greatly in IgE binding capacity of T cell response^37,55^. Other group 2 allergens such as Der f 2 and Lep d 2 have been also reported to have isoallergenic variants^56,57^. Similarly, in this study, nine isoforms of Blo t 2 have been identified. Polymorphic changes in Blo t 2 occur in 8 positions, with changes in position 96, 100 and 125 being the most frequent. In particular, polymorphisms involving G23, H29 and I96 may be implicated in changes in IgE reactivity, as these residues are located on the surface of the molecule, and the corresponding sequences were found to be important IgE epitopes in Der p 2^39–43^.

The panel of major allergens (defined as allergens that cause sensitization in >50% of the patients^58^) in *B. tropicalis* differs from that in *Dermatophagoides* spp. Specific IgE reactivity against *B. tropicalis* is mainly directed towards it’s group 5, 7 and 21 allergens, showing specific IgE reactivity to 40–50% of the population^10,45,50^, which is in contrast to *Dermatophagoides* spp. where groups 1, 2 and 23 dominate^59^. In our study, while specific IgE to Blo t 2 was only observed in a third of our population, this allergen remains clinically relevant as it has unique IgE epitopes that cannot be inhibited by group 2 allergens from *Dermatophagoides spp*. Future studies on the structure solution and detailed epitope mapping of Blo t 2 is needed to gain a better understanding on its cross reactive IgE epitopes. This would be particularly useful to generate hypoallergenic molecules devoid of the cross-reactive IgE epitopes that could be used for patients who are polysensitized to multiple group 2 allergens. Similar approaches in generation of hypoallergen candidates against other cross-reactive allergens have shown encouraging results^60,61^.

In summary, we report the identification and characterization of Blo t 2. This allergen is clinically relevant, with a 34% IgE binding within the Singaporean population. Importantly, as Blo t 2 has only limited cross-reactivity with Der p 2 and Der f 2, signifying the presence of unique epitopes. Hence, current diagnostic and therapeutic tools based on *Dermatophagoides* allergens may lead to under-diagnosis or ineffective treatment for individuals primarily sensitized by Blo t 2. Therefore, there is a need to include Blo t 2 in the diagnostic and therapeutic panels for precise and targeted diagnosis and treatment.

## Materials and Methods

### RT-PCR of putative Blo t 2 using degenerate primers

First strand cDNA was synthesised using SMART^TM^ RACE cDNA amplification kit (CLONTECH Laboratories, USA) with 1 µg *B. tropicalis* RNA as template. Degenerate primers designed based on the conserved regions of group 2 allergens (Blo t 2F, 5′-GMTGCCAACCARRACWC-3′ and Blo t 2R, 5′-CGCADGCBAANACACCRTKVTC-3′) were used to amplify a fragment of Blo t 2 with highly thermostable *pfu* polymerase. Based on the sequence amplified product, specific primers were designed and used for 5′ and 3′ random amplification of cDNA ends (RACE). The sequences of the RACE products were used to design primers to amplify the full-length DNA of Blo t 2. This product was then ligated to a modified pET-32a containing N-terminal hexa-histidine tag (Novagen) with T4 DNA ligase (Invitrogen). The plasmids were then transformed into the expression host, *Escherichia coli* BL21 (DE3).

### Ethics approval for serum samples and animal immunizations

Consecutive serum samples from Singaporean patients with clinical symptoms of allergies were used. Blood samples were obtained after getting written informed consent from all participants (n = 202, age range 13–55 years old, mean age = 24 years old). For participants under the age of 18 years, the parent and/or legal guardian provided the informed consent. Inclusion and exclusion criteria for selection for selecting patients for this study are detailed in Supplementary Table 3. Both human and animal studies were reviewed and approved by the Institutional Review Board (IRB) of the National Healthcare Group Domain Specific Review Board – B/04/055, National University of Singapore (NUS) IRB: 07-023, 09-256, 10-445, 13-075 and B14-150 and the Animal Research Ethics Committee of NUS and are in compliance with the Helsinki declaration.

### Cloning, expression, and purification of rBlo t 2

The Blo t 2 gene was cloned into a modified pET-32a (+) vector (pET-M) and overexpressed as a 6× His-tag fusion protein. The pET-M-Blo t 2 construct was transformed into the expression host *Escherichia coli* strain BL21 (DE3) to produce rBlo t 2. Transformed bacteria cells were grown overnight in Luria Bertani (LB, BioBasic, Inc.) containing ampicillin (100 µg/mL) at 37 °C. Overnight bacteria cultures were then inoculated and grown in 1L LB until OD_600_ reached 0.6. Recombinant protein expression was induced by the addition of 0.5 mM isopropyl β-D-1-thiogalactopyranoside (IPTG) to the bacterial cultures, which were grown at 37 °C for 4 h before harvesting by centrifugation at 5465 *g*. The pellet was resuspended in binding buffer (20 mM Tris–HCl pH 7.9, 0.5M NaCl, 20 mM imidazole, and 6M urea) and lysed by sonication at an amplitude of 38% for 15 min (30 s pulse on and 30 s pulse off) followed by centrifugation at 14 000 *g*. The supernatant containing the rBlo t 2 protein was filter-sterilized using a 0.22 µm PVDF membrane filter and then applied onto an Ni–NTA-coupled HisTrap HP 5 ml column (GE Healthcare, UK) which had been pre-equilibrated with 20 mL binding buffer. The rBlo t 2 protein was eluted using a linear gradient of washing buffer (20 mM Tris–HCl pH 7.9, 0.5M NaCl, 0.5M imidazole, and 6M urea). Fractions containing soluble rBlo t 2 protein were pooled and further purified by size-exclusion chromatography using a HiLoad 16/600 Superdex 75 pg gel-filtration column (GE Healthcare, UK) pre-equilibrated with refolding buffer (20 mM Tris–HCl pH 7.9 and 50 mM NaCl).

### Trypsin digestion

The Coomassie Brilliant Blue R-250-stained rBlo t 2 SDS–PAGE gel band was excised for protein identification. After washing thrice with 50 mM triethylammonium bicarbonate (TEAB) and 50% (v/v) acetonitrile (ACN), the finely-cut gels were dehydrated using 100% (v/v) ACN. To reduce the sample, 5 mM tris(2-carboxyethyl)phosphine) was added, followed by incubation at 57 °C for 1 h. After addition of 10 mM methyl methanethiosulfonate, the sample was kept at room temperature for 1 h. After reduction and alkylation, the gel pieces were washed with 50 mM TEAB. Then, the gel pieces were dehydrated using 100% (v/v) ACN and subsequently re-swelling was performed using 50 mM TEAB. A final dehydration was carried out using 100% (v/v) ACN. Trypsin (12.5 ng/µL) was then added and the samples were incubated at 37 °C for 16 h. The digested peptides were extracted sequentially using 50 mM TEAB, 5% (v/v) formic acid in 50% (v/v) ACN and 100% (v/v) ACN. These solutions were added and allowed to stand for 5–10 min before centrifuged at 3300 *g*. The supernatant with the digested peptides were collected after each extraction steps. Sample was lyophilized and then re-constituted in 2% (v/v) ACN containing 0.05% (v/v) formic acid. Before mass spectrometry, desalting was performed using Pierce^TM^ C18 Tips (Thermo Scientific). Sample was eluted in 80% (v/v) CAN containing 0.05% formic acid and vacuumed dried. Finally, the digested peptide sample was re-constituted in 2% (v/v) ACN containing 0.05% (v/v) formic acid.

### Mass spectrometric analysis

Peptide separation was performed using an Eksigent nanoLC Ultra and ChiPLC-nanoflex (Eksigent, Dublin, CA) in TrapElute configuration. Samples were loaded on a 0.5 mm × 200 μm column and eluted on an analytical 15 cm × 75 μm column (ChromXP C18-CL, 3 μm). A gradient formed by mobile phase A (2% (v/v) ACN, 0.1% (v/v) formic acid) and mobile phase B (98% (v/v) ACN, 0.1% (v/v) formic acid) was used to separate 2 μL of the sample. The flow rate was set at 0.3 μL/min. The following gradient elution was used for peptide separation: 0 to 5% of mobile phase B in 1 min, 5 to 12% of mobile phase B in 19 min, 12 to 30% of mobile phase B in 40 min, 30 to 90% of mobile phase B in 2 min, 90 to 90% in 7 min, 90 to 5% in 3 min and finally held at 5% of mobile phase B for 13 min. The tandem MS analysis was performed using a TripleTOF 5600 system (SCIEX) under Information Dependent Mode. For precursor ions selection, the mass range of 400–1800 m/z and accumulation times of 250 ms per spectrum were chosen. MS/MS analysis was performed on the 20 most abundant precursors with accumulation time of 100 ms per cycle. The dynamic exclusion time was at 15 s. High sensitivity mode with rolling collision energy was used to acquire the MS/MS spectra.

### Protein identification

Protein identification was carried out using ProteinPilot 5.0 software revision 4769 (SCIEX), which uses the Paragon database search algorithm (5.0.0.0.4767) and the integrated false discovery rate (FDR) analysis^62,63^ was applied to protein identification. The MS/MS spectra obtained were searched using the user-defined search parameters: (a) Cysteine alkylation: with MMTS; (b) Digestion: Trypsin; (c) Instrument: TripleTOF 5600; (d) ID focus: Biological modifications; (e) Search effort: Thorough; (f) FDR analysis: Yes; (g) User modified parameter files: Yes. The data was searched against the protein sequence database and downloaded from UniProtKB for Blo t 2 proteins on 24 April 2019 (total 559,235 entries). A decoy database search strategy was applied to estimate the FDR for peptide identification. A cut-off of unused protein score >1.3 (95% confidence) was applied for protein identification.

### Electrospray mass spectrometry and determination of disulfide bonds

Mass spectrometric detection of rBlo t 2 was performed using Synapt High Definition Mass Spectrometer (Manchester, UK) equipped with an electrospray ionization. The optimal operating conditions were as follows: the source temperature, 90 °C; desolvation gas temperature, 350 °C, cone gas flow, 40 L h^−1^; desolvation gas flow, 400 L h^−1^, capillary voltage, 3 kV; sampling cone voltage, 35 kV; extraction cone voltage, 3.5 kV. Sample was introduced with a continuum at a flow rate of 10 µL min^−1^, using Hamilton 250 µL syringe. The mass range acquired was from 100 to 250 Da with 1 s scan time over a 10 min run time. ProteoMass^TM^ Apomyoglobin MALDI-MS Standard (Sigma A8971) (16951Da) was used as the standard.

### Circular dichroism and polyclonal antibody production

Circular dichroism (CD) spectra was acquired using a J-810 Spectropolarimeter (Jasco, Japan) using a quartz cuvette with a path length of 1 mm. All experiments were conducted at room temperature, using 20 μM of protein in 50 mM sodium acetate pH 4.6. The spectra were recorded at the resolution of 0.1 nm with a scan speed of 50 nm/min and averaged for 10 scans from 190 to 260 nm. Polyclonal antibodies against Blo t 2 was raised in New Zealand White Rabbits by subcutaneous injection with 300 μg of recombinant protein using Freund’s adjuvant.

### Immuno-dot blot

Immuno-dot blot assays were performed as previously reported^64^. Serially diluted IgE standards (National Institute for Biological Standards, UK) and bovine serum albumin (BSA) were used as a positive and negative controls respectively. Intensities of each dot were measured using the Olympus Micromage^TM^ for Windows version 3.01 (Olympus Optical) image analysis software. IgE binding was classified based on optical density (OD) readings of the immuno dot blot. A reaction was classified as high (>100), medium (50–99), low (20–49) or negative (<20) from the maximum score of 255. All reactions with intensities > 20 (equivalent to 2SD above the mean of negative sera responses) were considered positive. Inter- and Intra-assay concordance were above 90% and 95% respectively, which demonstrated high assay reproducibility. Multiple dilution experiments were performed to demonstrate linear parallelism between the specific IgE and total IgE standard curves over the linear range of specific IgE dilutions.

### Inhibition ELISA

Maxisorp microtiter plates (Nunc) were coated overnight with 250 ng protein per well at 4 °C. Sera used were pre-absorbed with varied amounts of recombinant allergen. The plates were then washed three times with wash buffer (PBS-0.05% Tween-20) between each step of this assay. The plates were blocked for 1 hr with PBS-0.1% Tween 20 and incubated with pre-absorbed sera overnight at 4 °C. Biotin conjugated anti-human IgE monoclonal antibody (BD-Pharmingen, USA) was diluted 1:4000 in PBS and added to the wells for 2 hrs, followed by the addition of avidin conjugated HRP (BD-Pharmingen) diluted 1:3000 in PBS for 30 mins. The plates were washed six times, and 100 μL of 3,3′,5,5′-Tetramethylbenzidine (TMB) substrate was added to each well. The colorimetric reaction was stopped with 20 μL of 1M HCl, and absorbance measured at 450 nm using an ELISA plate reader. The degree of cross reactivity was calculated by the percentage of inhibition based on the following formula: [(I_u_ − I_i_)/(I_u_ − B)] × 100%, where I_u_ represents the reaction in the absence of inhibitor protein, I_i_ the reaction at a particular inhibitor concentration, and B represents background intensity when the allergen was incubated with blocking solution instead of sera.

### Isolation of Native Blo t 2

Native Blo t 2 was isolated using the Seize^TM^ Primary Immunoprecipitation Kit (Pierce) according to the manufacturer’s instructions. Briefly, rabbit polyclonal antibody-containing sera was coupled to the gel and incubated with 400 µL *B. tropicalis* crude protein extract. The natural protein was subsequently eluted 8 times with 400 µL elution buffer, and all the fractions were pooled and concentrated by lyophilization.

### Computer-based characterization and analysis

Amino acid sequences were analysed and aligned using either Clustal O (v1.2.4)^65,66^ or MEGA-X^67^ using the MUSCLE algorithm^68^. Prediction of the signal peptide cleavage site was done with the software SignalP v1.1^26^. N-glycosylation site prediction was performed online using NetNGlyc 1.0 Prediction Server at http://www.cbs.dtu.dk/services/NetNGlyc/21. Phylogenetic tree was generated by MEGA-X using the maximum-likelihood method with 1000 bootstrap.

### Histamine release assay

Histamine release in heparinized whole blood and histamine ELISA was performed using kits from IBL-Hamburg (Germany) according to the manufacturer’s protocol. Whole blood was collected from two dust-mite sensitized volunteers and three controls in heparinized tubes. Two hundred microliters of blood were then added to serial dilutions of Blo t 2 and incubated for 1 hr at 37 °C in a water bath. Histamine release was halted by incubating the samples for 10 mins in an ice bath. The samples were centrifuged for 10 mins at 700 *g*. 100 µL of supernatant was then used for Histamine-ELISA.

### Dust sample collection, processing and quantification

Dust samples were collected from kitchens, sofas, carpets and beds from Singaporean homes. Sampling was performed using a modified Kirby Classic III vacuum cleaner (Kirby Co.) adapted with a chamber that collects dust onto a filter paper. Each sample was obtained by vacuuming an area of 1 m^2^ for 2 mins. All dust samples were stored at −20 °C. The dust samples were sieved using a 500 μm pore size sieve. One milliliter of PBS was added for every 50 mg of dust sample, and allowed to shake overnight at 4 °C. The samples were then centrifuged at 2500 rpm for 20 mins at 4 °C, and the supernatant was stored at −20 °C.

### Measurement of amounts of allergen in dust samples using ELISA

One hundred microliters of individual dust sample were incubated overnight onto each well at 4 °C on monoclonal antibody coated microtitre plates (Maxisorp, Nunc) after the plates were blocked with 1% bovine serum albumin (BSA) in PBS for 30 mins at room temperature. The wells were then washed three times with PBS-0.05% Tween-20. The same washing method was used throughout the assay. The wells were incubated overnight with 100 μL of anti-Der f 2 or anti-Der f 22 IgG at 1:5000 dilutions in PBS at 4 °C. Wells were then washed and incubated with 1:1000 dilution of anti-rabbit IgG-conjugated horseradish peroxidase (BD-Pharmingen) in PBS for 3 hrs at room temperature. Wells were thoroughly washed before TMB (Sigma) was added. The reaction was stopped using 20 μL of 1 M HCl and plates were read at 450 nm. Allergen levels were quantified and denoted as microgram of allergen per gram of fine dust (µg/g).

### Isolation of Blo t 2 isoforms

Full length Blo t 2 was amplified from *B. tropicalis* cDNA using primers Blo t 2 LIC F: 5′-GACGACGACAAGATCATGTTCAAGTTTATCTGTCTC-3′ and Blot 2 LIC R: 5′-GAGGAGAAGCCCGGTTTAATCGACAACCTCGG-3′ with highly thermostable *pfu* polymerase. The PCR product was purified from a 1% agarose gel and annealed to pET-32(a) Ek/LIC Vector (Novagen) according to the manufacturer’s instructions. The recombinant pET-32(a) vectors were transformed into competent *E. coli* XL1-Blue cells (Stratagene). One hundred and forty colonies were picked, inoculated overnight and plasmid extracted. The plasmid DNA was then submitted to double pass sequencing, and all sequences were analysed using DNAMAN^®^ (Lynnon Corp., Quebec, Canada).

## Supplementary information

Supplementary Info

## References

[1] Resch Y (2015). Different IgE recognition of mite allergen components in asthmatic and nonasthmatic children. J Allergy Clin Immunol.

[2] Arlian LG, Platts-Mills TA (2001). The biology of dust mites and the remediation of mite allergens in allergic disease. J Allergy Clin Immunol.

[3] Chew FT, Lim SH, Goh DY, Lee BW (1999). Sensitization to local dust-mite fauna in Singapore. Allergy.

[4] Alimuddin S, Rengganis I, Rumende CM, Setiati S (2018). Comparison of Specific Immunoglobulin E with the Skin Prick Test in the Diagnosis of House Dust Mites and Cockroach Sensitization in Patients with Asthma and/or Allergic Rhinitis. Acta Med Indones.

[5] Yadav A, Naidu R (2015). Clinical manifestation and sensitization of allergic children from Malaysia. Asia Pac Allergy.

[6] Hussein AH, Elawamy W (2015). Quantitation of *Blomia tropicalis* allergen Blo t 5 in cereal and cereal-based foods consumed in the Nile Delta, Egypt. Am J Trop Med Hyg.

[7] Sanchez-Borges M, Capriles-Hulett A, Caballero-Fonseca F, Fernandez-Caldas E (2003). Mite and cockroach sensitization in allergic patients from Caracas, Venezuela. Ann Allergy Asthma Immunol.

[8] Panzner P (2018). Cross-sectional study on sensitization to mite and cockroach allergen components in allergy patients in the Central European region. Clin Transl Allergy.

[9] Stanaland BE, Fernandez-Caldas E, Jacinto CM, Trudeau WL, Lockey RF (1994). Sensitization to *Blomia tropicalis:* skin test and cross-reactivity studies. J Allergy Clin Immunol.

[10] Kuo IC (1999). Sensitization to *Blomia tropicalis* and *Dermatophagoides pteronyssinus*-a comparative study between Singapore and Taiwan. Asian Pac J Allergy Immunol.

[11] An S (2013). *Dermatophagoides farinae* allergens diversity identification by proteomics. Mol Cell Proteomics.

[12] Weghofer M (2013). Identification of Der p 23, a peritrophin-like protein, as a new major *Dermatophagoides pteronyssinus* allergen associated with the peritrophic matrix of mite fecal pellets. J Immunol.

[13] Chew FT (1999). Allergenic differences between the domestic mites *Blomia tropicalis* and *Dermatophagoides pteronyssinus*. Clin Exp Allergy.

[14] Gao YF (2007). Identification and characterization of a novel allergen from Blomia tropicalis: Blo t 21. J Allergy Clin Immunol.

[15] Tan KW (2012). NMR structure and IgE epitopes of Blo t 21, a major dust mite allergen from *Blomia tropicalis*. J Biol Chem.

[16] Carvalho Kdos A (2013). *Blomia tropicalis* Blo t 5 and Blo t 21 recombinant allergens might confer higher specificity to serodiagnostic assays than whole mite extract. BMC Immunol.

[17] Chua KY (1990). Isolation of cDNA coding for the major mite allergen Der p II by IgE plaque immunoassay. Int Arch Allergy Appl Immunol.

[18] Trudinger M, Chua KY, Thomas WR (1991). cDNA encoding the major mite allergen Der f II. Clin Exp Allergy.

[19] Varela J (1994). Primary structure of Lep d I, the main *Lepidoglyphus destructor* allergen. Eur J Biochem.

[20] Eriksson TL (1998). Cloning and characterisation of a group II allergen from the dust mite *Tyrophagus putrescentiae*. Eur J Biochem.

[21] Smith W, Mills K, Hazell L, Hart B, Thomas W (1999). Molecular analysis of the group 1 and 2 allergens from the house dust mite, *Euroglyphus maynei*. Int Arch Allergy Immunol.

[22] Gafvelin G (2001). Cross-reactivity studies of a new group 2 allergen from the dust mite *Glycyphagus domesticus*, Gly d 2, and group 2 allergens from *Dermatophagoides pteronyssinus, Lepidoglyphus destructor*, and *Tyrophagus putrescentiae* with recombinant allergens. J Allergy Clin Immunol.

[23] Ferrandiz R (1995). Characterization of allergenic components from house dust mite *Dermatophagoides siboney*. Purification of Der s 1 and Der s 2 allergens. Clin Exp Allergy.

[24] Altschul SF, Gish W, Miller W, Myers EW, Lipman DJ (1990). Basic local alignment search tool. J Mol Biol.

[25] Ghaffari-Nazari H (2015). Improving Multi-Epitope Long Peptide Vaccine Potency by Using a Strategy that Enhances CD4+ T Help in BALB/c Mice. PLoS One.

[26] Nielsen H, Engelbrecht J, Brunak S, von Heijne G (1997). Identification of prokaryotic and eukaryotic signal peptides and prediction of their cleavage sites. Protein Eng.

[27] Inohara N, Nunez G (2002). ML – a conserved domain involved in innate immunity and lipid metabolism. Trends Biochem Sci.

[28] Bateman A (2004). The Pfam protein families database. Nucleic Acids Res.

[29] Ichikawa S (1998). Solution structure of Der f 2, the major mite allergen for atopic diseases. J Biol Chem.

[30] Derewenda U (2002). The crystal structure of a major dust mite allergen Der p 2, and its biological implications. J Mol Biol.

[31] Gasteiger, E. *et al*. *Protein Identification and Analysis Tools on the ExPASy Server*. 571–607 (Humana Press, 2005). 10.1385/1-59259-584-7:531 10027275

[32] Ichikawa S (2005). NMR study on the major mite allergen Der f 2: its refined tertiary structure, epitopes for monoclonal antibodies and characteristics shared by ML protein group members. J Biochem.

[33] Johannessen BR (2005). Structure of the house dust mite allergen Der f 2: implications for function and molecular basis of IgE cross-reactivity. FEBS Lett.

[34] Suzuki M, Tanaka Y, Korematsu S, Mikami B, Minato N (2006). Crystal structure and some properties of a major house dust mite allergen, Derf 2. Biochem Biophys Res Commun.

[35] Lin RY (2000). Histamine and tryptase levels in patients with acute allergic reactions: An emergency department-based study. J Allergy Clin Immunol.

[36] Smith AM (2001). Sequence polymorphisms and antibody binding to the group 2 dust mite allergens. Int Arch Allergy Immunol.

[37] Park JW (2002). Der p 2 isoallergens have different allergenicity, and quantification with 2-site ELISA using monoclonal antibodies is influenced by the isoallergens. Clin Exp Allergy.

[38] Christensen LH, Riise E, Bang L, Zhang C, Lund K (2010). Isoallergen variations contribute to the overall complexity of effector cell degranulation: effect mediated through differentiated IgE affinity. J Immunol.

[39] Ipsen H (2004). Mapping of Der p 2 antibody binding epitopes by site directed mutagenesis. Journal of Allergy and Clinical Immunology.

[40] Reginald K, Chew FT (2018). Conformational IgE Epitope Mapping of Der p 2 and the Evaluations of Two Candidate Hypoallergens for Immunotherapy. Sci Rep.

[41] Mueller GA, Smith AM, Chapman MD, Rule GS, Benjamin DC (2001). Hydrogen exchange nuclear magnetic resonance spectroscopy mapping of antibody epitopes on the house dust mite allergen Der p 2. J Biol Chem.

[42] Smith AM, Chapman MD (1997). Localization of antigenic sites on Der p 2 using oligonucleotide-directed mutagenesis targeted to predicted surface residues. Clin Exp Allergy.

[43] Hakkaart GA, Aalberse RC, van Ree R (1998). Epitope mapping of the house-dust-mite allergen Der p 2 by means of site-directed mutagenesis. Allergy.

[44] Reginald K, Chew FT (2019). The major allergen Der p 2 is a cholesterol binding protein. Sci Rep.

[45] Kidon MI (2011). Mite component-specific IgE repertoire and phenotypes of allergic disease in childhood: the tropical perspective. Pediatr Allergy Immunol.

[46] Posa D (2017). Evolution and predictive value of IgE responses toward a comprehensive panel of house dust mite allergens during the first 2 decades of life. J Allergy Clin Immunol.

[47] Galli SJ, Tsai M (2012). IgE and mast cells in allergic disease. Nat Med.

[48] Meno KH, Kastrup JS, Kuo IC, Chua KY, Gajhede M (2017). The structure of the mite allergen Blo t 1 explains the limited antibody cross-reactivity to Der p 1. Allergy.

[49] Kuo IC, Cheong N, Trakultivakorn M, Lee BW, Chua KY (2003). An extensive study of human IgE cross-reactivity of Blo t 5 and Der p 5. J Allergy Clin Immunol.

[50] Soongrung T (2018). The *Blomia tropicalis* allergen Blo t 7 stimulates innate immune signalling pathways through TLR2. Clin Exp Allergy.

[51] Kim CR (2015). Crossreactivity between group-5 and -21 mite allergens from *Dermatophagoides farinae*, *Tyrophagus putrescentiae* and *Blomia tropicalis*. Mol Med Rep.

[52] Lau S (1989). High mite-allergen exposure increases the risk of sensitization in atopic children and young adults. J Allergy Clin Immunol.

[53] Arruda LK (1991). Exposure and sensitization to dust mite allergens among asthmatic children in Sao Paulo, Brazil. Clin Exp Allergy.

[54] Smith AM (2001). The molecular basis of antigenic cross-reactivity between the group 2 mite allergens. J Allergy Clin Immunol.

[55] Hales BJ, Hazell LA, Smith W, Thomas WR (2002). Genetic variation of Der p 2 allergens: effects on T cell responses and immunoglobulin E binding. Clin Exp Allergy.

[56] Olsson S (1998). Expression of two isoforms of Lep d 2, the major allergen of *Lepidoglyphus destructor*, in both prokaryotic and eukaryotic systems. Clin Exp Allergy.

[57] Jeong KY (2012). Sequence polymorphisms of Der f 1, Der p 1, Der f 2 and Der p 2 from Korean house dust mite isolates. Exp Appl Acarol.

[58] Lowenstein H (1978). Quantitative immunoelectrophoretic methods as a tool for the analysis and isolation of allergens. Prog Allergy.

[59] Raulf M (2015). Mites and other indoor allergens - from exposure to sensitization and treatment. Allergo J Int.

[60] Swoboda I (2002). Mutants of the major ryegrass pollen allergen, Lol p 5, with reduced IgE-binding capacity: candidates for grass pollen-specific immunotherapy. Eur J Immunol.

[61] Neudecker P (2003). Mutational epitope analysis of Pru av 1 and Api g 1, the major allergens of cherry (*Prunus avium*) and celery (*Apium graveolens*): correlating IgE reactivity with three-dimensional structure. Biochem J.

[62] Shilov IV (2007). The Paragon Algorithm, a next generation search engine that uses sequence temperature values and feature probabilities to identify peptides from tandem mass spectra. Mol Cell Proteomics.

[63] Tang WH, Shilov IV, Seymour SL (2008). Nonlinear fitting method for determining local false discovery rates from decoy database searches. J Proteome Res.

[64] Reginald K (2018). Characterization of Der f 22 - a paralogue of the major allergen Der f 2. Sci Rep.

[65] Sievers F (2011). Fast, scalable generation of high-quality protein multiple sequence alignments using Clustal Omega. Mol Syst Biol.

[66] Sievers F, Higgins DG (2018). Clustal Omega for making accurate alignments of many protein sequences. Protein Sci.

[67] Kumar S, Stecher G, Li M, Knyaz C, Tamura K (2018). MEGA X: Molecular Evolutionary Genetics Analysis across Computing Platforms. Mol Biol Evol.

[68] Edgar RC (2004). MUSCLE: multiple sequence alignment with high accuracy and high throughput. Nucleic Acids Res.

[69] Waterhouse A (2018). SWISS-MODEL: homology modelling of protein structures and complexes. Nucleic Acids Res.

[70] Pettersen EF (2004). UCSF Chimera–a visualization system for exploratory research and analysis. J Comput Chem.

