# Supplementary material for: Blo t 2: Group 2 allergen from the dust mite Blomia tropicalis
Source: Sci Rep. 2019 Aug 22;9:12239. doi: 10.1038/s41598-019-48688-y (PMC6706440; doi:10.1038/s41598-019-48688-y)
Supplement: Supplementary file 1 — Supplementary Info [file 41598_2019_48688_MOESM1_ESM.pdf]

Blo t 2: Group 2 allergen from the dust mite *Blomia tropicalis*

Short title: Characterization of Blo t 2 allergen

Reginald Kavita[1#, 2], Pang Sze Lei[2] and Chew Fook Tim[2]*

[1]Department of Biological Sciences, Sunway University, Bandar Sunway 47500, Selangor, Malaysia

[2]Allergy and Molecular Immunology Laboratory, Department of Biological Science, National University of Singapore, 117543 Singapore

[#]Current address

Corresponding author:

Dr. Chew Fook Tim, Allergy and Molecular Immunology Laboratory, Lee Hiok Kwee Functional Genomics Laboratories, Department of Biological Sciences, 14 Science Drive 4, National University of Singapore, 117543 Singapore, Tel. +65-65161685; Fax +65-67792486; E-mail: dbscft@nus.edu.sg

Supplementary Table 1. Identified proteins based on tryptic digested peptides of rBlo t 2 using the TripleTOF 5600 mass spectrometry.

N[1]	Unused[2]	Total[3]	%Cov[4]	Accession	Name	Species	Peptides (95%)[5]
1	91.1	91.1	95.8		Mite allergen Blo t 2	*Blomia tropicalis*	369
25	2	80.21	85.9	tr|A6XEP1|A6XEP1_BLOTA	Group 2 allergen Blo t 2 isoform 4 OS=Blomia tropicalis OX=40697 PE=2 SV=1	*Blomia tropicalis*	324
26	2	80.21	85.9	tr|A6XEP6|A6XEP6_BLOTA	Group 2 allergen Blo t 2 isoform 9 OS=Blomia tropicalis OX=40697 PE=2 SV=1	*Blomia tropicalis*	324
27	2	79.57	85.9	tr|Q1M2P2|Q1M2P2_BLOTA	Type 2 allergen Blo t 2.046 OS=Blomia tropicalis OX=40697 PE=2 SV=1	*Blomia tropicalis*	324
57	0.36	78.41	84.5	tr|A6XEP4|A6XEP4_BLOTA	Group 2 allergen Blo t 2 isoform 7 OS=Blomia tropicalis OX=40697 PE=2 SV=1	*Blomia tropicalis*	323
8	7.26	71.06	85.9	tr|A6XEP3|A6XEP3_BLOTA	Group 2 allergen Blo t 2 isoform 6 OS=Blomia tropicalis OX=40697 PE=2 SV=1	*Blomia tropicalis*	278

[1]N: the rank of the specified protein relative to all other proteins in the detected list

[2]Unused Protscore/ Unused protein score: a measure of the protein confidence for a detected protein, calculated from the peptide confidence for peptides from spectra that are not already completely "used" by higher scoring winning proteins.

[3]Total ProtScore/ Total protein score: a measure of the total amount of evidence for a detected protein. The Total ProtScore is calculated using all of the peptides detected for the protein. The Total ProtScore does not indicate anything about the confidence that a protein has been detected, because some or even all of the spectra contributing to the Total ProtScore may be better explained by higher ranked proteins.

[4]% Cov (coverage): The percentage of matching amino acids from identified peptides having confidence greater than 0% divided by the total number of amino acids in the sequence.

[5]Peptides (95%): The number of distinct peptides that have at least 95% confidence. Multiple modified and cleaved states of the same underlying peptide sequence are considered as distinct peptides because they have different molecular formulas. Multiple spectra of the same peptide due to replicate acquisition or different charge states are counted only once.

Supplementary Table 2. Published IgE Epitopes of Der p 2

Mutation	Ref.	Method	Results
K6A	39	site directed mutagenesis	marginal reduction in IgE binding
N10A	40	Serum IgE binding	IgE binding <75% compared to WT Der p 2 in at least 40% of population (n=116)
K15A	39	site directed mutagenesis	significant reduction in IgE binding
		mAb binding	mAb binding abolished
E25A	40	Serum IgE binding	IgE binding <75% compared to WT Der p 2 in at least 40% of population (n=116)
H30A	41	mAb binding (7A1)	ΔΔ G > 0.50 kcal/mol
R31A	41	mAb binding (7A1)	ΔΔ G > 0.50 kcal/mol
K33A	41	mAb binding (7A1)	ΔΔ G > 0.50 kcal/mol
S57A	41	mAb binding (7A1)	ΔΔ G > 0.50 kcal/mol
E62A	39	site directed mutagenesis	marginal reduction in IgE binding
C73R	42	mAb binding (alpha DpX)	14% inhibition compared to WT Der p 2
		mAb binding (15E11)	<2% inhibition compared to WT Der p 2
		mAb binding (13A4)	13% inhibition compared to WT Der p 2
		serum inhibition	17% inhibition with pooled sera from 7 dust-mite allergic patients
H74A	39	site directed mutagenesis	significant reduction in IgE binding
		mAb binding	mAb binding abolished
K77A	40	Serum IgE binding	IgE binding <75% compared to WT Der p 2 in at least 40% of population (n=116)
K82A	39	site directed mutagenesis	significant reduction in IgE binding
K96A	41	mAb binding (7A1)	ΔΔ G > 0.50 kcal/mol
	40	Serum IgE binding	IgE binding <75% compared to WT Der p 2 in at least 40% of population (n=116)*
I97A	40	mAb binding (7A1)	ΔΔ G > 0.50 kcal/mol
K100A	42	mAb binding (13A4)	<10% inhibition compared to WT rDer p 2
		mAb binding (7A1)	30% inhibition for mutant K100T compared to WT Der p 2
E102A	41	mAb binding (7A1)	ΔΔ G > 0.50 kcal/mol
	40	Serum IgE binding	IgE binding <75% compared to WT Der p 2 in at least 40% of population (n=116)
Δ22-26	43	mAb binding (10E11, alpha DpX, 7A1)	reduced binding to 3 mAb tested
		RAST inhibition - patient sera	10-1000 fold reduction in IgE binding to 6 patient sera tested
C73A.C78A.Δ74-77	43	mAb binding (2B12B3)	5-fold reduction in mAb binding (RAST inhibition)
		mAb binding (alphaDpX)	30-10 fold weaker inhibitor compared to WT Der p 2 (RAST)
		RAST inhibition - patient sera	Variable reduction (minimal - 100 fold) in IgE binding to 6 patient sera tested
N44P.Q45P.N46H	42	mAb binding (13A4, alpha DpX, 15E11)	<10% inhibition compared to WT rDer p 2
		serum inhibition	40% inhibition with pooled sera from 7 dust-mite allergic patients

[39] Ipsen et al 2004

[40] Reginald and Chew 2018

[41] Mueller et al 2001

[42] Smith and Chapman 1997

[43] Haakart et al 1998

* This mutant was reported to be misfolded

Supplementary Table 3. Inclusion and exclusion criteria for patients used in this study

Inclusion criteria	
	Singaporean citizen
	Doctor diagnosed allergy and/or allergic rhinitis
	Positive skin prick results and/ or had IgE binding to dust-mite crude protein extracts (*Dermatophagoides spp.* and/or *Blomia tropicalis*) as tested using immuno-dot blots
Exclusion criteria	
	Not a Singaporean citizen
	Negative skin prick results and/ or had IgE binding to dust-mite crude protein extracts (*Dermatophagoides spp.* and/or *Blomia tropicalis*) as tested using immuno-dot blots

```
                     1            2     3
Blo t 2      -GDVKFTDCAHGEVTSLDLSGCSG-DHCTIHKGKSFTLKTFFIANQDSEKLEIKISATMN   58
Der p 2      RDQVDVKDCANHEIKKVLVPGCHGSEPCIIHRGKPFQLEAVFEANQNTKTAKIEIKASID   60
              .:*...***: *:..: : ** * : * **:** * *::.* ***:::. :*:*.*:::

                          4    5                                        6
Blo t 2      GIEVPVPGVDKDGCKHTTCPLKKGQKYELDYSLIIPTILPNLKTV-TTASLVGDHGVVAC   117
Der p 2      GLEVDVPGIDPNACHYMKCPLVKGQQYDIKYTWNVPKIAPKSENVVVTVKVMGDDGVLAC   120
             *:** ***:* :.*:: .*** ***:*::.*:  :*.* *: :.* .*..::**.**:**

Blo t 2      GKVNTEVV-D  126
Der p 2      AIATHAKIRD  130
             . ..   : *
```

Supplementary Figure 1. Alignment of mature proteins of Blo t 2 (GenBank ID ABG76185) and Der p 2 (AAF86462) was performed using Clustal O (v1.2.4). An asterisk (*) indicated the positions with fully conserved residues, a colon (:) indicated conservation between groups of strongly similar properties, and a period (.) indicated conservation between groups of weakly similar properties. Numbers above the sequences indicate the presence of a cysteine residue in any of the proteins.

```
                                 1                    2          3
Blo t 2    1  -GDVKFTDCAHGEVTSLDLSGC-SG-DHCTIHKGKSFTLKTFFIANQDSEKLEIKISATMNG-IEVPVPGVDKD  70
Gly d 2    1  -GKMNFTDCGHNEIKELSVSNC-TGNY-CVIHRGKPLTLDAKFDANQDTASVGLVLTAIIDGDIAIDIPGLETN  71
Lep d 2    1  -GKMTFKDCGHGEVTELDITGC-SG-DTCVIHRGEKMTLEAKFAANQDTAKVTIKVLAKVAG-TTIQVPGLETD  70
Pso c 2    1  -GKVKFQDCGKGEVESLEVEGC-SG-DYCVIHKGKKLDLAISVTSNQDSANLKLDIVADING-VQIEVPGVDHD  70
Sui m 2    1  -GEMKFQDCGHGEVKKLLVSDC-SG-DYCIIHKGKKLSMEADFVANQDSPTAVIKISAKVNG-VELQVPGIETN  70
Ale o 2    1  -GQVRFEDCGHHEVTKLDISQCADGASTCVIHKGKELKLDAEILANQDSAKIEVHLTANIDG-LSIPIPGVDKD  72
Aca s 2    1  --ELKFKDCGHHEVTKVVVNDC-DGAY-CVLHKSKPVNFAATFVANQDSAKLHLEVLGSLNG-LTIPVPGVPSD  69
Tyr p 2    1  -GQVKFTDCGKKEIASVAVDGC-EG-DLCVIHKSKPVHVIAEFTANQDTCKIEVKVTGQLNG-LEVPIPGIETD  70
Der f 2    1  -DQVDVKDCANNEIKKVMVDGC-HGSDPCIIHRGKPFTLEALFDANQNTKTAKIEIKASLDG-LEIDVPGIDTN  71
Der s 2    1  -DQVDVKDCANNEIKKVMVDGC-HGSDPCIIHRGKPFTLEALFDANQNTKTAKIEIKANIDG-LEVDVPGIDTN  71
Der p 2    1  RDQVDVKDCANHEIKKVLVPGC-HGSEPCIIHRGKPFQLEAVFEANQNTKTAKIEIKASIDG-LEVDVPGIDPN  72
Eur m 2    1  -DQVDIKDCANHEIKKVMVPGC-KGSEPCVIHRGTAFQLEAVFDANQNSNAAKIEIKATIDG-VEIDVPGIDNN  71
LEPDE2     1  -KDIKFKDCANGELKSVDVDPC-D-SDPCVFKKGRPVTLKAKFVANHNVENVELKATIDLEG-IDIKYPGLDSN  70

                4     5                                          6
Blo t 2   71  GCKHTT-CPLKKGQKYELDYSL-IIPTILPNLKTVT-TASLV--GD-HGVVA-CG-KVNTEVVD  126
Gly d 2   72  ACKLM-KCPIRKGEHQELIYNIGEIPDATPE-K-AKVKAQLI--GE-HGVLA-CG-WVDGEVQE  127
Lep d 2   71  GCKFI-KCPVKKGEALDFIYS-GTIPAITPKVK-ADVTAELI--GD-HGVMA-CG-TVHGQV-E  125
Pso c 2   71  GCHYV-KCPIKKGQHFDVKYTY-SIPAILPTTK-AKIIAKII--GD-KGL-GGCI-VINGEIQD  126
Sui m 2   71  GCHHM-KCPLVKGQSYQFKYDL-VIPQILPNVK-ADVTASLT--GA-HGLLA-CG-TVHGEVQN  126
Ale o 2   73  GCKYVT-CPIKKGEKVHFNYSL-VVPKLIPNLHNVEVGAKIT--GD-HGLLA-CL-RLKGDIAN  129
Aca s 2   70  GCKVV-KCPLVKGQTYTAKYSM-NIPSIIPVTKSVV-TVKLT--GD-HGVVA-CG-SVDGEIAA  125
Tyr p 2   71  GCKVL-KCPLKKGTKYTMNYSV-NVPSVVPNIK-TVVKLLAT--GE-HGVLA-CG-AVNTDVKP  126
Der f 2   72  ACHFM-KCPLVKGQQYDIKYTW-NVPKIAPKSENVVVTVKLI--GD-NGVLA-CAIATHGKIRD  129
Der s 2   72  ACHFI-KCPLVKGQQYDAKYTW-NVPKIAPKSENVVVTVKLI--GD-NGVLA-CAIATHAKIRD  129
Der p 2   73  ACHYM-KCPLVKGQQYDIKYTW-NVPKIAPKSENVVVTVKVM--GDD-GVLA-CAIATHAKIRD  130
Eur m 2   72  LCHFM-KCPLVKGQEYDIKYTW-NVPRIAPKSENVVVTVKLL--GD-NGVLA-CAIATHAKIRD  129
LEPDE2    71  GCNYLS-CPLVQGKEYELVYKV---PVMQGLAD-MRSSMKWVLTGKETRKVLGCA-EVEFEVQS  128
```

Supplementary Figure 2. Multiple alignment of mature proteins of group 2 allergens and homologous proteins from thirteen different dust mite species using MEGA-X. Genbank IDs for protein sequences used were Blo t 2 (ABG76185), Gly d 2 (Q9U5P7), Lep d 2 (P80384), Pso c 2 (AQQ10895), Sui m 2 (AAS75831), Ale o 2 (AAS75832), Aca s 2 (ABU97459), Tyr p 2 (CAA73221), Der f 2 (Q00855), Der s 2 (ABC96702), Der p 2 (AAF86462), Eur m 2 (Q9TZZ2) and LEPDE2 (RWS27379). The group 2 allergen homologue from *Leptotrombidium delience* was named as LEPDE2 for simplicity. Multiple alignment was performed using the MUSCLE algorithm. Identical aligned sequences were coloured based on the Clustal-X colour coding by amino acid properties; hydrophobic (A, I, L, M, F, W, V) -blue, positively charged (K, R) -red, negatively charged (E, D) – magenta, polar (N, Q, S, T) – green, cysteines (C) – pink, glycines (G) – orange, prolines (P) – yellow, aromatic (H, Y) – cyan. Unconserved residues are coloured white. Numbers above the sequences indicate the presence of a cysteine residue in any of the proteins.

A)

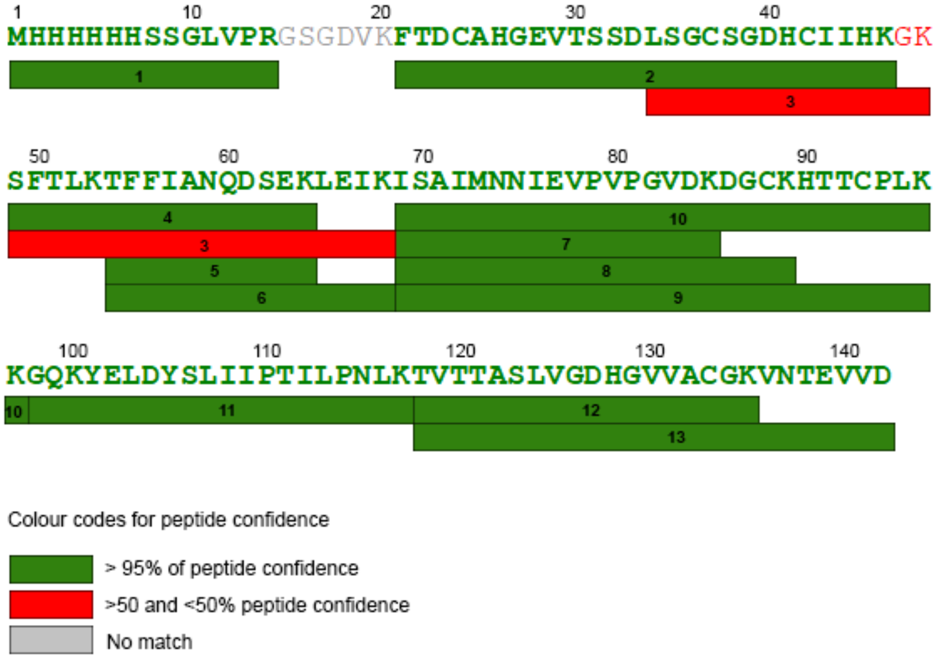

B)

Tryptic fragment	Residue	Sequence	Theoretical mass	Experimental mass
1	1-14	MHHHHHHSSGLVPR	417.96	417.96
2	21-46	FTDCAHGEVTSSDLSGCSGDHCIIHK	715.04	715.03
3	34-48	LSGCSGDHCIIHKGKSFTLKTFFIANQDSEK LEIK	997.50	995.98
4	49-64	SFTLKTFFIANQDSEK	625.99	625.99
5	54-64	TFFIANQDSEK	650.31	650.31
6	54-68	TFFIANQDSEKLEIK	905.96	905.95
7	69-85	ISAIMNNIEVPVPGVDK	906.48	906.46
8	69-89	ISAIMNNIEVPVPGVDKDGCK	743.04	743.04
9	90-97	ISAIMNNIEVPVPGVDKDGCKHTTCPLK	615.10	615.10
10	90-98	ISAIMNNIEVPVPGVDKDGCKHTTCPLKK	800.89	800.65
11	98-117	GQKYELDYSLIIPTILPNLK	773.44	773.44
12	118-135	TVTTASLVGDHGVVACGK	587.63	587.63
13	118-142	TVTTASLVGDHGVVACGKVNTEVVD	848.09	848.11

Supplementary Figure 3. (A) Matched peptide sequences of rBlo t 2 obtained using the TripleTOF 5600 (Applied Biosystems-SCIEX) mass spectrometer. Protein sequence coverage is 95.8%. The identified sections of sequences are colour-coded. Peptides identified with high confidence (>95%) are in green, while peptides with low confidence (between 0-50%) are in red. Peptides in grey indicate portions of sequences with no spectral evidence. (B) Tryptic fragments and the corresponding mass value for rBlo t 2 peptide sequences. Cysteine residues of rBlo t 2 are underlined.

A

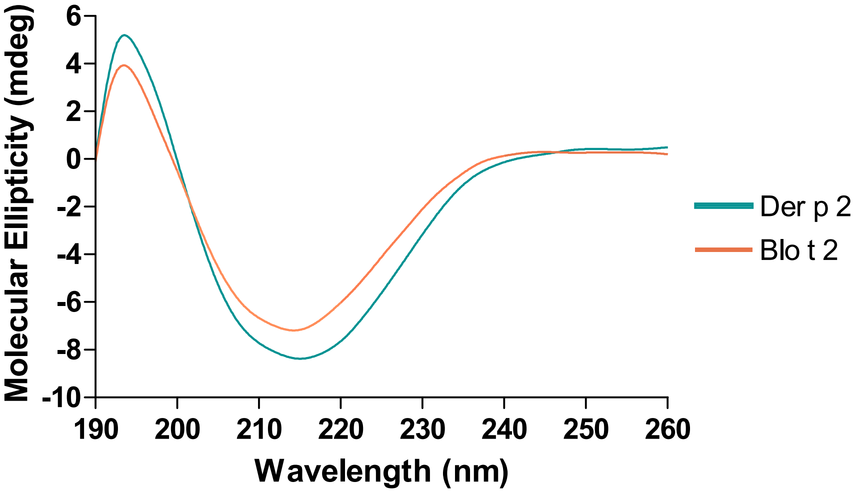

B

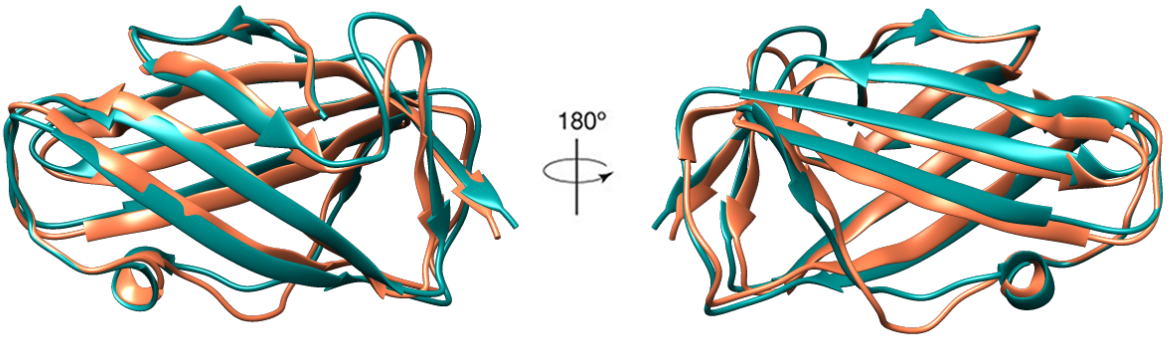

Supplementary Figure 4. (A) Far UV circular dichroism (CD) spectra of recombinant Blo t 2 and Der p 2. Twenty micromolar of each protein, in 50 mM sodium acetate pH 4.6 was used to obtain the CD spectra. Blo t 2 showed a typical β-sheeted protein spectrum similar to Der p 2. Average spectra from 10 scans are shown. (B) Superimposition of Blo t 2 model (coral) and Der p 2 (PDB code 1KTJ) (cyan) shows root-mean-square deviation (r.m.s.d) of 1.45 Å over 123 Cα atoms. The figure was generated using the Chimera program.

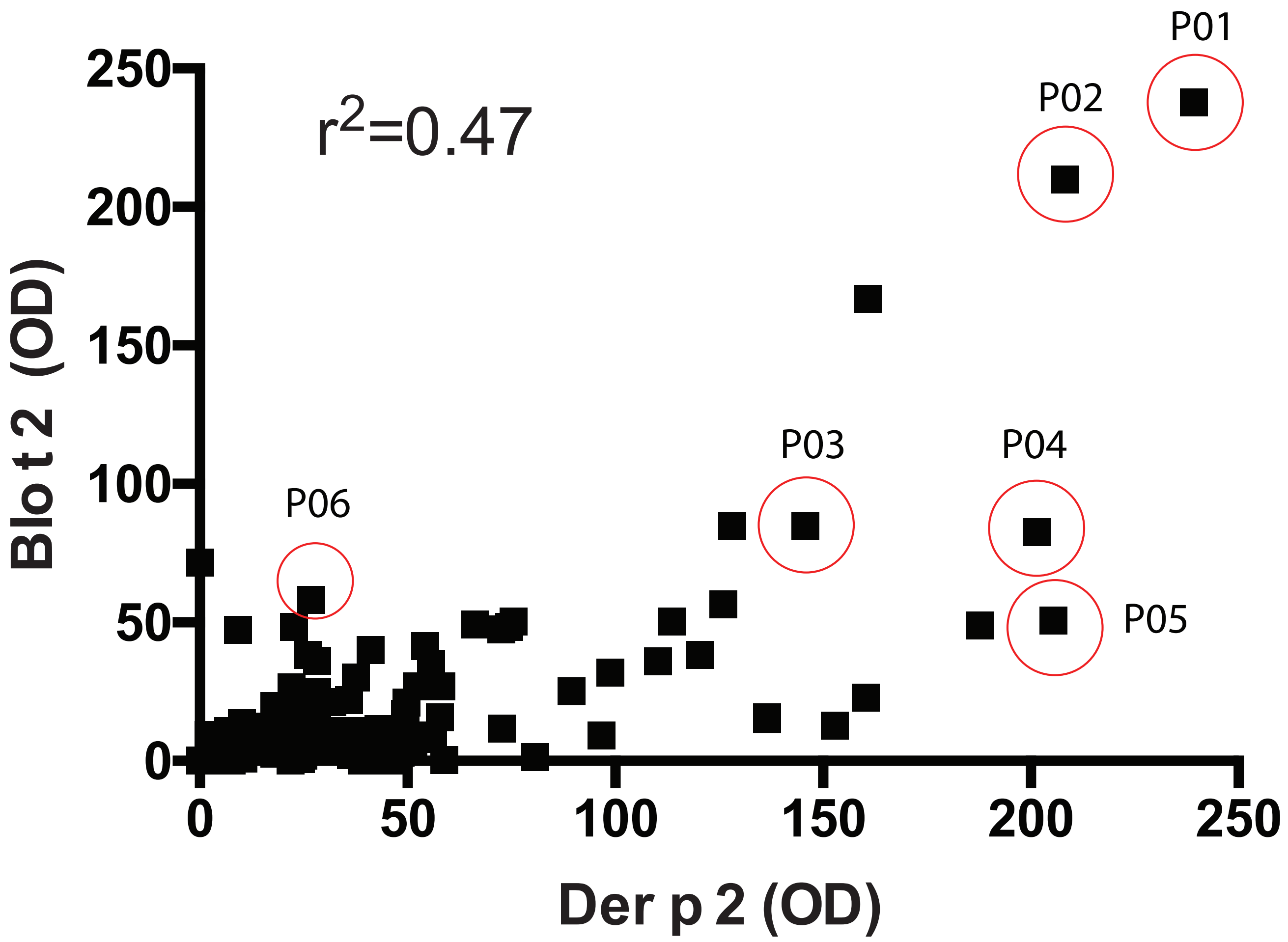

Supplementary Figure 5. Dot-plot of IgE-binding of atopic patients' sera to Der p 2 and Blo t 2, showing the IgE-binding intensities and correlation between allergens ($p < 0.0001$). The individual patients (P01-P06) used in IgE-inhibition studies are circled.

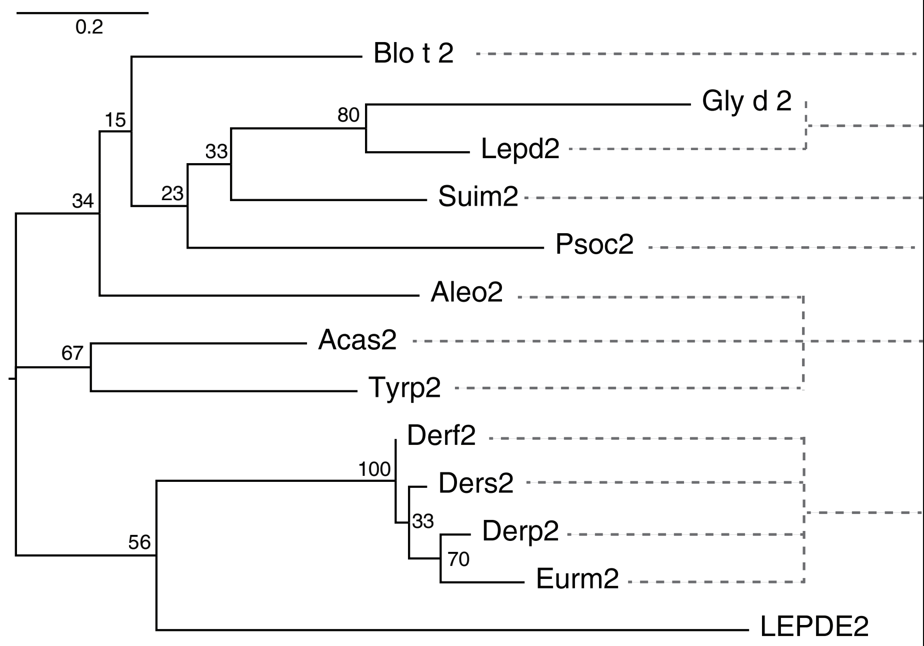

**Family**	**Superfamily**	**Suborder**	**Order**	**Suborder**
Echimyopodidae	Glycyphagoidea	Astigmata	Sarcoptoformes	Acariformes
Glycyphagidae				
Suidasiidae	Acaroidea			
Psoroptidae	Sarcoptoidea			
Acaridae	Acaroidea			
Pyroglyphidae	Analgoidea			
Trombiculidae	Trombiculoidea	Prostigmata	Trombidiformes	

Supplementary Figure 6. Phylogenetic relationships of group 2 allergens and homologous proteins from thriteen dust mite species. Mature protein sequences were used for alignments. The phylogenetic tree was generated by MEGA-X using the maximum-likelihood method. Numbers on the branches indicate bootstrap values (1000 simulations). Accession numbers of sequences used were similar to that in Supplementary Figure 2. Corresponding taxonomy of each species is provided.

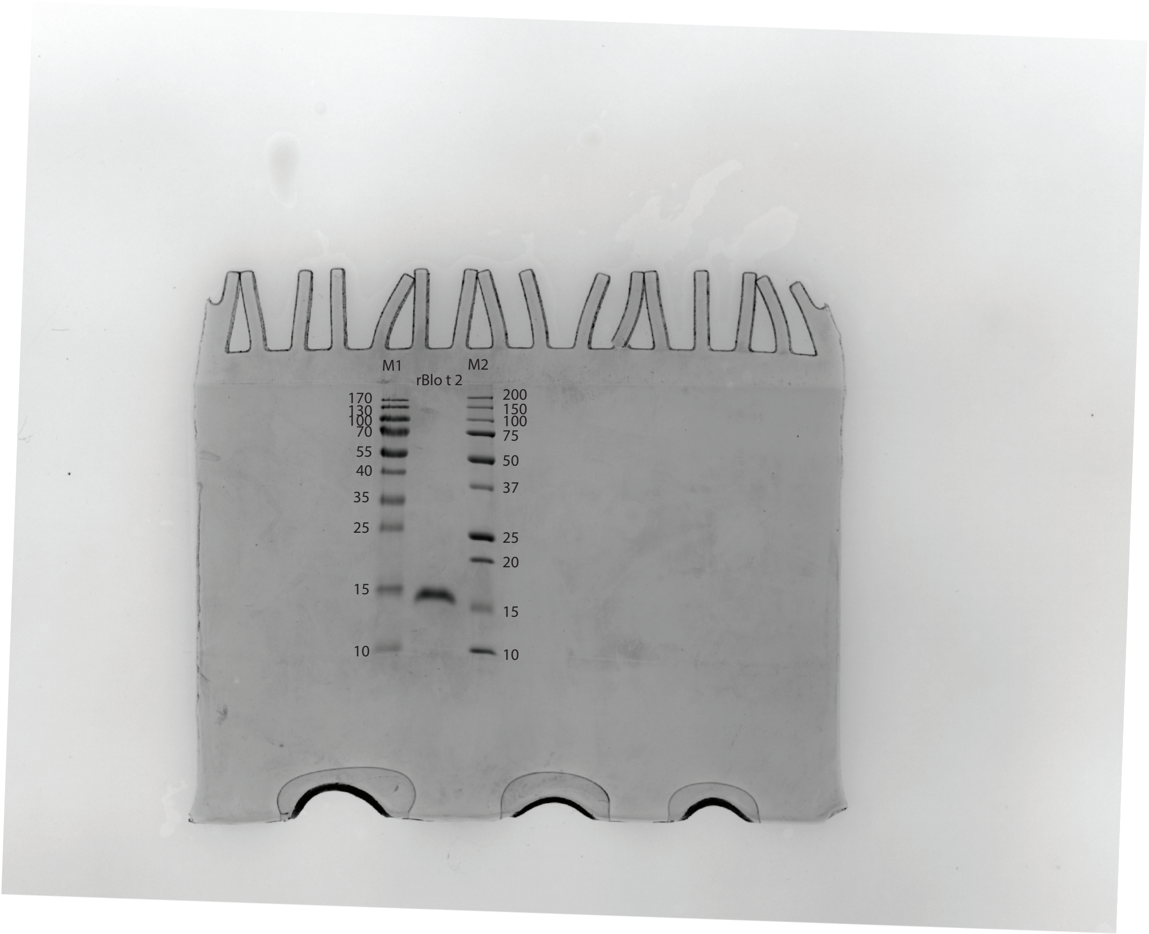

Supplementary Figure 7. Analysis of purified recombinant Blo t 2 (rBlo t 2). Purified rBlo t 2 and two different molecular mass markers (M1 and M2) was separated by 15% SDS-PAGE and stained with Coomasie brilliant blue. Molecular weights are in kilo Daltons (kDa). Full SDS-PAGE gel is presented.
